# Host Mitochondrial Requirements of Cytomegalovirus Replication

**DOI:** 10.1007/s40588-020-00153-5

**Published:** 2020-09-30

**Authors:** Chandler H. Monk, Kevin J. Zwezdaryk

**Affiliations:** 1 Department of Microbiology & Immunology, Tulane University Health Sciences Center, 1430 Tulane Ave #8638, New Orleans, LA 70112, USA

**Keywords:** Mitochondria, CMV, ETC, Membrane potential, Oxidative phosphorylation, ROS

## Abstract

**Purpose of Review:**

Metabolic rewiring of the host cell is required for optimal viral replication. Human cytomegalovirus (HCMV) has been observed to manipulate numerous mitochondrial functions. In this review, we describe the strategies and targets HCMV uses to control different aspects of mitochondrial function.

**Recent Findings:**

The mitochondria are instrumental in meeting the biosynthetic and bioenergetic needs of HCMV replication. This is achieved through altered metabolism and signaling pathways. Morphological changes mediated through biogenesis and fission/fusion dynamics contribute to strategies to avoid cell death, overcome oxidative stress, and maximize the biosynthetic and bioenergetic outputs of mitochondria.

**Summary:**

Emerging data suggests that cytomegalovirus relies on intact, functional host mitochondria for optimal replication. HCMV large size and slow replication kinetics create a dependency on mitochondria during replication. Targeting the host mitochondria is an attractive antiviral target.

## Introduction

As obligate parasites, viruses are dependent on the host for replication. By themselves, they are incapable of energy production. Key to this is a viral dependency on the host cellular metabolic network for replication. Nearly 70 years ago, it was established that many viruses including poliomyelitis virus, herpes simplex virus 1, and Rous sarcoma virus altered glycolytic pathways of infected cells [1–3]. These observations were expanded to include manipulation of adenosine triphosphate (ATP) production and RNA breakdown. Through altered host metabolism, viruses can support their mass production. This requires manipulation of host organelles and resources to reproduce viral particles. Biosynthetic (nucleotides, amino acids, lipids) and bioenergetic requirements are critical for viral replication. Many of the metabolic pathways targeted or altered during viral infection converge or are dependent on the host mitochondria.

**Fig. 1:**
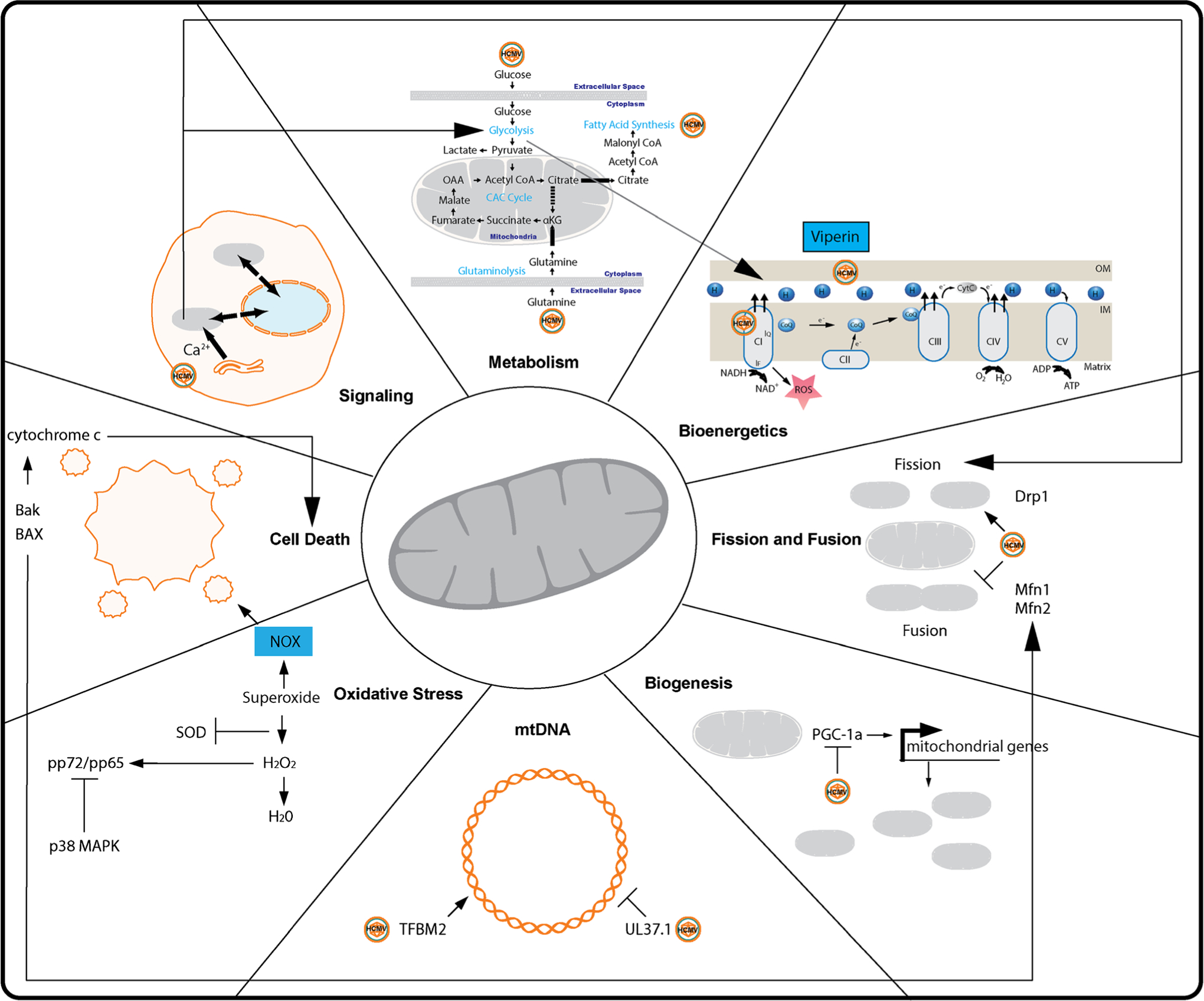
Overview of mitochondria and HCMV. Interactions with mitochondrial metabolism, bioenergetics, fission/fusion, biogenesis, mtDNA, oxidative stress, cell death, and signaling are shown as described in text

Mitochondria have long been regarded as the powerhouse of the cell. Yet, this organelle has been shown to have essential functions in signal transduction pathways, cellular metabolism, immune response, cell cycle, and cell death (reviewed in [4]) (Fig. 1). The mitochondrion is composed of outer and inner membranes. The outer membrane surrounds the inner membrane space, an area that contains many apoptotic factors (e.g., cytochrome complex (cyt-c), endonuclease G). These factors are released by apoptotic signaling cascades received by the mitochondria. The inner membrane is a folded structure termed cristae that is responsible for transport of water, gases, and protein complexes required for electron transport chain (ETC) function. Within the inner membrane is the matrix, which contains molecules required for energy production and mitochondrial function. ATP is generated using oxidative phosphorylation (OXPHOS) by transferring electrons produced by the citric acid cycle (CAC) through the mitochondrial respiratory complexes. By hijacking the mitochondria, viruses can control the whole cell.

Human cytomegalovirus (HCMV) depends on many aspects of mitochondrial function for efficient viral replication. HCMV is a large double-stranded DNA betaherpesvirus. Likely due to its slow growth and vast coding potential, HCMV utilizes the host mitochondria to meet the bioenergetic and biosynthetic requirements of viral replication [5]. Metabolic profiling of HCMV-infected cells confirmed elevated glycolytic and pyrimidine nucleotide syntheses and suggested increased rates of the citric acid cycle [6••]. Long-chain fatty acid synthesis and lipid metabolism have since been shown to be targeted by HCMV [7, 8]. Interestingly, HCMV-induced metabolic alterations are very similar to metabolism observed in many tumor cells.

The specific focus of this review is to explore the role of the host mitochondria during HCMV replication. We highlight key mitochondrial pathways targeted by HCMV, discuss the mechanisms by which HCMV alters host mitochondrial function, and reflect on how this impacts bio-energetic, biosynthetic, and mitochondrial morphology pathways that benefit HCMV.

## Mitochondrial Biosynthetic and Bioenergetic Pathways

### Citric Acid Cycle

HCMV infection alters numerous metabolic pathways of host cells including glycolysis, glutaminolysis, and lipid synthesis [6••, 9, 10]. A common feature of these pathways is an interaction or dependency on the host mitochondria, specifically trafficking through the CAC. HCMV strong induction of glycolysis results in increased glucose-derived citrate that is exported from the mitochondria and used to support fatty acid synthesis [6••, 10]. Glutaminolysis is upregulated, possibly through manipulation of c-Myc activity, to maintain activation of the CAC [9]. The viral protein U_L_38 was shown to aid in glucose and glutamine upregulation by inhibiting tuberous sclerosis complex 2 (TSC2) [11]. TSC2 negatively regulates mammalian target of rapamycin (mTOR) complex I, thus acting as a metabolic sensor with a key role in glucose transport. These studies all emphasize how HCMV rewires host metabolic pathways, promoting elevated carbon flux through key metabolic pathways. A recent study used the Seahorse bioanalyzer to record live cell, real-time measurements showing increased glycolysis and mitochondrial function during HCMV infection, validating these previous observations [12]. Precisely how HCMV alters these major metabolic pathways is not completely understood, but recent publications have illuminated the role of carbohydrate regulatory element–binding proteins (ChREBPs) and sterol regulatory element–binding proteins (SREBPs) as key transcription factors targeted by HCMV [13, 14].

### Oxidative Phosphorylation/Electron Transport Chain

The generation of NADH and FADH_2_ in the CAC provides electrons used to reduce oxygen to water and the generation of ATP through the process of OXPHOS. HCMV has been noted to interact directly with and alter function of ETC complexes. Munger et al. show that transcription of ETC-related enzymes and the ATP-synthesizing proton pump are upregulated in HCMV-infected cells [6••]. Viral mitochondria-localized inhibitor of apoptosis (vMIA), a protein encoded by HCMV UL37, suppresses phosphate transport through binding to the ETC ATPase inorganic phosphate carrier (PiC), resulting in decreased levels of ATP [15, 16]. Reeves et al. proved that HCMV 2.7-kb RNA transcript (β2.7) colocalizes with ETC complex I [17••]. Through this interaction, the mitochondrial membrane potential was stabilized, allowing ATP production to be uninterrupted. Kairney et al. observed an increase in OXPHOS as well as subparts of complex IV; however, they did not find a significant increase in complex II [18•]. In agreement, a recent paper observed an increase in protein levels of ETC complex subunits II, III, and IV, and ATPase after HCMV infection [12]. Another study focused on all genes related to OXPHOS and reported that all 24 genes related to the ETC were upregulated during HCMV infection [19]. Lastly, to emphasize the importance of the ETC to HCMV replication, Combs et al. knocked down mitochondrial DNA (mtDNA) in fibroblasts [12]. The mtDNA is critical for coding proteins of the ETC subunits. The mtDNA knockdown cells have functional mitochondria but no ETC function. HCMV viral titers were significantly inhibited in the mtDNA knockdown cells. HCMV immediate-early (IE) expression was unaffected, but early and late viral protein expressions were delayed and decreased. This is a strong indicator of HCMV dependency on a highly functional OXPHOS/ETC system for robust, efficient viral replication.

### NAD+/NADH

Nicotinamide adenine dinucleotide (NAD) is critical to provide cells with a mechanism to accept and donate electrons specifically during OXPHOS. A dramatic increase in NADH levels during HCMV infection has been reported [6••]. This is likely due to increased demand rather than a reduced consumption level of NADH. The increase in glycolysis during infection may contribute to elevated acetyl-CoA and NADH levels [6••]. Anaplerotic-associated increases in NADH, using glutamate dehydrogenase (GDH) to measure glutaminase activity, have been observed [9]. Despite indirect measurement of NADH, changes are inferred, as it is a product of GDH and can be used as a marker for glutaminase and GDH levels. During HCMV infection, glutaminase and glutamate dehydrogenase levels doubled in infected cells [9]. Lactate dehydrogenase (LDH) reduction of pyruvate to lactate also increased ~25 fold [9]. In this case, NADH is converted to NAD+, revealing that the NAD+/NADH ratio is variable dependent on the CAC cycle [9]. The current literature suggests that NAD+/NADH levels vary during HCMV infection, but due to limited studies, many questions remain unanswered. The development of novel tools to study NAD levels real time in live cells may provide insight into the dependency of NAD during HCMV infection.

### Membrane Potential

The mitochondrial membrane potential, coupled with the proton gradient, is responsible for the transmembrane potential of hydrogen ions required for production of ATP. Membrane potential and ATP levels must remain stable, as prolonged changes can promote mitochondrial damage. Pioneering studies by Landini et al. established that HCMV infection altered membrane potential in HCMV-infected human embryo fibroblasts [20]. They observed hyperpolarization of infected cells late during infection (42 h post-infection; hpi), suggesting that mitochondrial alterations occur as infection persists [20]. A recent paper validated these findings by showing significant elevation of mitochondrial membrane potential beginning at 48 hpi [12]. Other reports suggest that hyperpolarization of the mitochondrial membrane potential occurs upon HCMV infection and is reliant on viral-induced upregulation of glycolysis [21]. Lower levels of cyt-c release suggested that increased mitochondrial membrane potential is an anti-apoptotic mechanism driven by expression of vMIA [21]. Calcium efflux from the endoplasmic reticulum to the mitochondria is mediated by vMIA, upregulating glycolysis, resulting in increased mitochondrial membrane potential [21]. Additionally, β2.7 interaction with ETC complex I has been shown to stabilize mitochondrial membrane potential during infection [17••]. This interaction prevented relocalization of an apoptosis mediator, thus inhibiting cell death. Differences in mitochondrial membrane potential were noted when using the β2.7 knockout HCMV strain, ΔToledoβ2.7. Infection with ΔToledoβ2.7 resulted in a significant decrease in mitochondrial membrane potential. These results implicate the importance of HCMV β2.7 in stabilizing mitochondrial membrane potential through direct interactions with ETC complex I [17••].

Alternatively, studies have shown depolarization of the mitochondrial membrane during infection [22, 23]. The HCMV glycoprotein US9 was reported to localize and disrupt the mitochondrial membrane, reducing the association between translocase of outer membrane (TOM) 20 and TOM70 through competitive binding [23, 24]. This results in a loss of membrane potential and disruption of the membrane’s structural integrity, allowing leakage of the mitochondrial antiviral signaling protein (MAVS) from the outer membrane [23]. This has also been observed with hepatitis C, resulting in MAVS cleavage from the outer membrane [25]. Depolarization of the mitochondrial membrane and subsequent disruption of MAVS have been suggested to be a viral immune evasion tactic [25]. Lee et al. report that HCMV infection disrupts mitochondrial membrane potential as early as 24 hpi [26]. Depolarization drives release of cyt-c and induction of apoptosis, allowing efficient release of replicated virus from dying cells [26]. Decreased membrane potential also triggers a cyt-c antiviral response [26]. HCMV also stimulates production of an antiviral protein, viperin, which translocates to the mitochondrial outer membrane with assistance from vMIA. The viperin/vMIA association and translocation are necessary for disruption of mitochondrial membrane permeability and potential [15, 27]. Decreased mitochondrial membrane potential results in lower ATP levels and mitochondrial fission, observed as highly fragmented mitochondria [15, 22, 28, 29]. These mechanisms are possible viral strategies to promote release of viral progeny. Early inhibition of apoptosis may allow HCMV to efficiently replicate, while promotion of apoptosis at later time points could benefit the virus by enhancing viral release.

The vast discrepancies in the literature are likely due to a combination of factors. Without parallel data on mitochondrial biogenesis during HCMV infection, it is difficult to rigorously define changes to mitochondrial membrane potential [20, 30]. Similarly, iron chelation can affect membrane potential as has been suggested during HCMV infection [22]. The time point at which measurements are recorded may also impact results. Ideally, measurements would be taken across the entire replication cycle of the virus. Currently, the varying results and proposed mechanisms regarding mitochondrial membrane potential of HCMV-infected cells provide an incoherent picture of why, when, and how HCMV alters mitochondrial membrane potential.

### ATP

Despite continued characterization of viral hijacking of host metabolic systems, the energetic costs of viral replication remain poorly understood [31]. All viruses require energy in the form of ATP to replicate. Under normal cellular conditions, ATP is predominantly generated by the ETC. During conditions of metabolic stress, ATP can be generated quicker, but less efficiently, through glycolysis. Upregulation of glycolysis during HCMV infection has been well documented (reviewed in [32, 33]). It has been suggested that increased glycolytic capacity is needed to meet the energetic and/or biosynthetic requirements of HCMV replication. Interestingly, few studies have observed changes to host ATP levels during HCMV infection.

Numerous groups have reported no significant changes to ATP levels during infection [6••, 12, 17••, 22]. The viral long non-coding RNA β2.7 was shown to maintain ATP levels during infection [17••]. Using the β2.7 KO strain ΔToledoβ2.7, ATP levels were significantly decreased during infection. Chambers et al. report a substantial increase in ATP during infection with glutamate supplementation [34]. Alternatively, decreased ATP levels have been reported. Viperin interaction with the mitochondrial trifunctional protein, mediated by vMIA, decreases cellular ATP levels [27]. vMIA-expressing cells were found to inhibit phosphate carrier activity inhibiting ATP generation [15]. Reduced ATP levels were observed in both HeLa and NIH3T3 cells stably expressing vMIA. How a protein that is localized on the outer mitochondrial membrane might exert control over a carrier located in the inner mitochondrial membrane is unknown.

Conflicting data exists on the role of ATP production during HCMV infection. Using a predictive model, it was estimated that influenza replication uses 1% of the total energy available in a eukaryotic cell [31]. By analogy, HCMV infection would utilize a fraction of the total energy available with a host cell.

### Reactive Oxygen Species

Reactive oxygen species (ROS) produced by ETC complexes can be beneficial or deleterious to viral replication. Low levels of ROS have been shown to alter signaling pathways [35]. High ROS concentrations are toxic to the cell. Early papers indicated an accumulation of intracellular ROS during HCMV infection. Speir et al. observed that ROS scavengers have a deleterious effect on HCMV gene expression, suggesting that ROS upregulation is advantageous for HCMV replication [36]. ROS can activate nuclear factor-kappa B (NF-κB), which has several binding sites within the HCMV major immediate-early promoter (MIEP) [36–38]. Elegant studies demonstrated that NF-κB is required for HCMV MIEP transactivation [39]. Further studies revealed that cyclooxygenase-2 (COX-2), which is regulated by NF-κB, plays a role in increased ROS levels stimulated by HCMV [38]. Not surprisingly, levels of ROS are reported to increase as HCMV infection persists [12, 40]. Addition of sirtinol, a sirtuin inhibitor, to infected cells decreased ROS levels possibly through SirT2 and P16^NK4^ activation [40]. Murine CMV (MCMV) upregulation of ROS is correlated with inflammasome activation and nucleotide-binding oligomerization domain-like receptor protein 3 (NLRP3) pathway [41]. As ROS levels increase during MCMV infection, the NLRP3 inflammasome is activated, leading to inflammation and neurological defects that are associated with MCMV [41]. Inhibition of ROS pathways significantly decreased NLRP3-associated inflammation, suggesting that HCMV upregulation of ROS is linked to pathology [41]. Elevated superoxide levels and the decreased activity of the antioxidant superoxide dismutase (SOD) further strengthen observations of elevated ROS pathways during infection [12, 41].

HCMV-induced ROS production may also function as an immune evasion tactic [42]. Intracellular ROS increases via the NOX complex and initiates cell death mechanisms through the parthanatos pathway [42]. This may also be inducing apoptosis of immune cells in the microenvironment [42]. The cytotoxic mechanism of ROS had been reported previously with T cells and is linked to mitochondria, but the role of HCMV to this pathway is novel [42, 43].

ROS has also been implicated in the activation of the viral replication process. Xiao et al. evaluated the impact of H_2_O_2_ on the replication cycle of HCMV [44]. Elevated H_2_O_2_ concentrations promoted upregulation of viral transcription and protein expression of HCMV pp72 and pp65. The addition of H_2_O_2_ scavengers decreased HCMV replication. These effects are likely mediated through phosphorylation of p38 mitogen–activated protein kinase (p38 MAPK), as targeting the MAPK pathway increased ROS levels and increased expression of viral proteins such as IE [45].

This data suggests that antioxidants and associated treatments targeting ROS could prove to be a valuable antiviral treatment for HCMV infections. Viral replication levels decreased when applying cyclophilin A (CyPA) to decrease H_2_O_2_-mediated p38 MAPK activation [44]. Tilton et al. suggest that HCMV employs mechanisms to clear ROS and superoxide from the cell based on detection of increased levels of glutathione and SOD in HCMV-infected cells [46]. Glutathione inhibition decreased viral titers at early time points post-infection, but the efficacy waned over time [46]. HCMV may encourage expression of select antioxidants to prevent oxidative stress–related signaling pathways (e.g., mTOR) and maintain cellular homeostasis [46]. Scholz et al. performed a similar study in which they also inhibited glutathione; however, they found that the resultant oxidative stress was accompanied with a rise in HCMV replication and viral protein production [47]. Strong data supports increased elevated metabolic activity during HCMV infection. This logically suggests that during replication, elevated OXPHOS and ETC activities will eventually result in the generation of high levels of ROS. The current data suggest that initial low levels of ROS may benefit HCMV replication, but as levels continually increase, antioxidant mechanisms fail, and toxic concentrations of ROS contribute to cell death.

## Mitochondrial Morphological Changes

### Mitochondrial Biogenesis and Turnover

Mitochondrial biogenesis occurs in response to increased demand for mitochondrial metabolic capacity. Multiple groups have reported increased mtDNA synthesis during HCMV infection; however, the extent varies widely. Increases in mtDNA synthesis of 3-fold to 300-fold have been reported [12, 18•, 30, 34]. Increased expression of transcription factor B2 mitochondria (TFB2M), a mitochondrial transcription factor, was observed within 24 hpi, displaying significant increases at 48 hpi [18•]. This suggests that HCMV IE expression may be involved in increased mtDNA synthesis [18•]. A second study observed that increases in mtDNA synthesis and mtDNA copy numbers during HCMV infection could be reduced using a *UL37x1* knockout HCMV strain [34]. Additionally, lack of pUL37 (vMIA) resulted in decreased viral titers, and the production of new mitochondria diminished [34].

Synthesis of mtDNA is an indicator of ensuing mitochondrial biogenesis. Therefore, an increase in mtDNA replication supports data showing that mitochondrial biogenesis is increased in HCMV-infected cells [18•, 22, 34]. Mitoribosomal biogenesis is substantially increased in HCMV-infected cells and proves to be advantageous for viral propagation. Blocking of mitochondrial translation using chloramphenicol results in reduced viral titers [18•]. Crowe et al. observed “grain-like” mitochondria in HCMV-infected cells, suggesting increased mitochondrial biogenesis [22]. Similarly, the presence of mitochondrial rRNA methyltrans-ferase 3 (MRM3) was used as a marker for mitochondrial biogenesis. Protein expression showed a dramatic upregulation of MRM3 in HCMV-infected cells as early as 24 hpi, suggesting that HCMV infection promoted mitochondrial biogenesis [18•]. A polymerase inhibitor, phosphonoformic acid, was used to block expression of viral genes expressed late during the infection cycle, and no impact on MRM3 or TFB2M levels was observed [18•]. Together, this suggests that early or immediate-early viral gene expression is altering MRM3 and TFB2M expressions and not viral late gene expression. These observations were also seen at the mRNA level. The mitochondrial protein, peroxisome proliferator–activated receptor gamma co-activator 1 (PGC-1α), was targeted as a possible mechanism promoting mitochondrial biogenesis. PGC-1α is a master regulator of mitochondrial biogenesis. PGC-1α is expressed at significantly higher rates in wild type HCMV-infected cells as compared with *UL37x1* knockout strains, suggesting that infection is inducing mitochondrial biogenesis [34]. HCMV also causes an increase in mitochondrial mass, which is independent of pUL37 (vMIA) activity [34].

Hertel et al. employed a different approach by focusing on mitochondrial genes expressed at late times of infection. They reported that mitochondria-related genes are expressed at significantly higher levels in infected cells. Over 90% of the assessed mitochondria-related genes were upregulated during HCMV infection. Some upregulated genes were specific to biosynthesis of mitochondrial components, such as the ETC [19]. Weekes et al. also observed similar gene expression related to mitochondrial pathways, matching their observations of increased expression of OXPHOS and fatty acid synthesis pathways [48]. In general, mitochondria-associated genes upregulated during infection are associated with mitochondrial enzymes and proteins. Altered gene expression has been reported to occur as early as 12 hpi [18•].

### Mitochondrial Fission and Fusion

Fragmented mitochondria are typically observed during HCMV infection [15, 22, 49, 50]. Using Sendai virus and encephalomyocarditis virus as a model, Koshiba et al. illustrated that inhibition of the mitochondrial fusion proteins, Mitofusin 1 (Mtf1) and Mitofusin 2 (Mtf2), disrupts antiviral signaling [25]. Viral infections can disrupt the mitochondrial fusion process, resulting in fragmented mitochondria, without inducing cellular dysfunction.

During HCMV infection, vMIA has been observed to induce mitochondrial fragmentation [50, 51•]. HCMV vMIA deletion mutants are unable to induce fragmentation, suggesting that vMIA directly or through an unknown pathway promotes fragmentation [49]. vMIA recruits Drp1 to the mitochondria through Ca^2+^ flux [52, 53]. Drp1 is a key component of fission regulation and activation; therefore, HCMV relocating and utilizing Drp1 could aid the virus in creating widespread fragmentation of the mitochondria [52–54]. It has also been suggested that this mechanism may contribute to inhibition of apoptosis [53].

The Bcl-2 family members, Bax and Bak, and their role in mitochondrial elongation are also exploited during HCMV infection [55]. Bax induces the assembly of Mfn2. Double knockout Bax and Bak cells fail to induce mitochondrial fusion, leading to fragmented mitochondria [55]. Poncet et al. found that vMIA’s ability to cause a segmented mitochondrion morphology is unrelated to the anti-apoptotic interaction with Bax and likely related to metabolism [15, 51•]. They note that vMIA is known to recruit the apoptosis-inducing protein Bax to the mitochondria, causing a disruption to the outer mitochondrial membrane permeability, possibly contributing to fission of the mitochondria. The change in permeability could be attributed to vMIA preventing Bax from binding to adenine nucleotide translocator (ANT) and forming channels or a direct interaction between ANT and vMIA. The mechanism is unidentified, and it is also unknown if apoptosis is simply prevented by early fission events or if these are separate and distinct actions of vMIA [49].

Despite structural similarities between vMIA and Bcl-2, it has been noted that there are differences that may affect function [51•]. Binding of vMIA with GADD45 family members, which usually bind with Bcl-2 family members, has been reported [56]. An association between vMIA and GADD45α may form a complex with Bcl-x_L_, leading to increased levels of vMIA protein expression and punctate mitochondria [56]. A decrease in total mitochondria is not observed, just size, showing that despite fission being upregulated, it is not resulting in significantly increased rates of mitophagy, as would be expected [56]. This provides evidence that vMIA has a motif that is structurally similar to the Bcl-2 family and may manipulate Bcl-2-associated pathways [51•, 56].

It has been suggested that PiC may be a mediator for vMIA-mediated fragmentation of mitochondria [15, 50, 51•, 57]. The recruitment of Bax in this outcome appears to be unrelated, as Bax knockdown still results in fragmentation. This suggests that vMIA is regulating mitochondrial fission through a mechanism unrelated to its anti-apoptotic function [57]. Inhibition of PiC in the presence of vMIA has been observed to decrease metabolic products such as ATP [15]. It has also been shown that PiC acts as a permeability regulator of the mitochondrial membrane [57]. It remains unknown whether PiC downregulation causes fission as a result of metabolic changes or through changing the permeability of the inner mitochondrial membrane [57].

In regard to fusion, in non-infected cells, it is observed that mitochondrial membrane potential, and more specifically inner mitochondrial membrane potential, is vital for fusion to occur [28, 29]. Decreased mitochondrial membrane potential increases fission and decreases fusion, resulting in punctate mitochondria [29]. HCMV effects on this process are discussed earlier in this review.

## Conclusions

Mitochondria are critical for HCMV replication. To date, there are only a few identified viral products that specifically target host mitochondria (Table 1). Deeper study will likely reveal new HCMV candidates that are employed to manipulate mitochondrial function. HCMV utilization of the host mitochondria is likely due to its size, complexity, and long replication cycle. HCMV has a large genome and encodes a vast protein arsenal prepackaged in the tegument and on the surface. Lastly, there are strenuous lipid requirements for both new progeny and cytomegaly (swelling of the infected host cell). The mitochondria are central in providing the building blocks and energy required for this to occur, all while inhibiting cell death. It is interesting to speculate on the short- versus long-term effects of HCMV infection on the mitochondria. Many of the studies referenced in this review employed an acute infection model. This provides insights into HCMV-mediated mitochondrial reprogramming or dysfunction, but cannot address long-term functional changes to the host mitochondria. This may be critical missing data as HCMV has been associated with many age-related diseases, many of which included mitochondrial dysfunction as hallmarks of the disease. Inclusion of chronic infection models that accurately mimic in vivo conditions may provide novel metabolic mechanisms linking HCMV infection to age-related disease initiation or progression. By teasing out the mechanisms of mitochondrial manipulation during HCMV infection, we can learn about the intricacies and limits of mitochondria.

## Figures and Tables

**Table 1:** Overview of mitochondrial interactions

Mitochondrial change	Viral mechanism	Host pathway targeted	Potential consequences	Source
Increased membrane potential	vMIA	PFK	↑ calcium efflux, ↑ membrane potential, ↑ glycolysis	[12, 20,21]
Decreased membrane potential	US2, US9, vMIA	HFE, TOM20, TOM70, MAVS, Bax, and cyt-c	↑ cytomegaly, ↑ labile iron pool, ↑ mitochondrial fission, ↓ TOM70, ↓ membrane potential, ↑ cyt-c	[22, 23, 26]
Unchanged membrane potential	Beta 2.7	ETC complex I	↓ apoptosis	[17••]
Increased ROS	MIE, IE72, IE84	H2O2, p38-MAPK, SirT1, p16NK4, NOX-2, SOD, NLRP3 inflammasome, NF-κB, COX-2, HLA, GSH	↑ oxidative stress, ↑ senescence, ↑ inflammasome	[12, 26, 38, 40, 41, 44, 47]
Decreased ROS	Unknown	Glutathione, Nrf2, ATF6	↓ oxidative stress	[46]
Increased NADH	Unknown	PFK-1	↑ OXPHOS, ↓ oxidative stress	[9, 10]
Increased fission	vMIA	Bak, Bax, PiC, GADD45alpha	↑ fission, ↓ membrane potential	[12, 15, 29, 50, 51•, 56]
Decreased ATP	vMIA	PiC, ANT	Unknown	[15]
Increased ATP	Unknown	Unknown	Unknown	[9]
Unchanged ATP	Unknown	Unknown	Unknown	[12, 22]
